# Improved Methods for Treatment of Phytopathogenic Biofilms: Metallic Compounds as Anti-Bacterial Coatings and Fungicide Tank-Mix Partners

**DOI:** 10.3390/molecules24122312

**Published:** 2019-06-22

**Authors:** Michael Harding, Patricia Nadworny, Brenton Buziak, Amin Omar, Greg Daniels, Jie Feng

**Affiliations:** 1 Alberta Agriculture and Forestry, Crop Diversification Centre South, 301 Horticulture Station Road East, Brooks, AB T1R 1E6, Canada; greg.daniels@gov.ab.ca; 2 Innovotech, Inc., Suite L131, 2011-94 Street, Edmonton, AB T6N 1H1, Canada; Brenton.buziak@innovotech.ca (B.B.); amin.omar@innovotech.ca (A.O.); 3 Alberta Agriculture and Forestry, Alberta Plant Health Lab, 17507 Fort Road NW, Edmonton, AB T5Y 6H3, Canada; jie.feng@gov.ab.ca

**Keywords:** Oxysilver nitrate, pentasilver hexaoxoiodate, silver, copper, white mold, bacterial blight, *Sclerotinia sclerotiorum*, *Pseudomonas*, *Xanthomonas*

## Abstract

Fungi and bacteria cause disease issues in cultivated plants world-wide. In most cases, the fungi and bacteria colonize plant tissues as biofilms, which can be very challenging to destroy or eradicate. In this experiment, we employed a novel (biofilm) approach to crop disease management by evaluating the efficacies of six fungicides, and four silver-based compounds, versus biofilms formed by fungi and bacteria, respectively. The aim was to identify combinations of fungicides and metallic cations that showed potential to improve the control of white mold (WM), caused by the ascomycete fungus *Sclerotinia sclerotiorum*, and to evaluate novel high valency silver compounds as seed coatings to prevent biofilm formation of four bacterial blight pathogens on dry bean seeds. Our results confirmed that mature fungal biofilms were recalcitrant to inactivation by fungicides. When metallic cations were added to the fungicides, their efficacies were improved. Some improvements were statistically significant, with one combination (fluazinam + Cu^2+^) showing a synergistic effect. Additionally, coatings with silver compounds could reduce bacterial blight biofilms on dry bean seeds and oxysilver nitrate was the most potent inhibitor of bacterial blight.

## 1. Introduction

Biofilms are multicellular communities of microorganisms attached to solid surfaces and encased in a self-produced polymeric matrix (EPS) [1]. Biofilms are formed by bacteria, yeast, filamentous fungi, and oomycetes, and are known to respond to, or be influenced by, a number of factors in the surrounding environment such as substrate, nutrients, metabolites, elements, co-factors, and other signaling molecules [2,3]. For example, metal cations are frequently involved in, or essential for, vital physiological and metabolic cellular functions. Some metallic cations have been reported to enhance biofilm formation [3], while others have inhibitory or anti-biofilm properties [4]. One of the first interactions described for metal ions and biofilms was the binding of metals by the EPS [5]. More recently, the inhibition of biofilms by metal cations has been intensively studied revealing the powerful effects of these cations and their potential use in inhibition and remediation of biofilms [6,7,8,9]. Some of the cations are directly toxic or antagonistic [10], while others may interfere with signaling [11]. 

Bacterial and fungal biofilm formation in crop disease is well-documented [12,13,14,15,16]. A biofilm approach to management of citrus canker was reported where inhibition of biofilms formed by *Xanthomonas citri* subsp. *citri* was observed with d-leucine and 3-indolylacetontrile [17]. These inhibitory treatments made the pathogen more susceptible to copper-based bactericide treatment. However, despite this example, a biofilm approach in agricultural microbiology has been extremely rare. This situation leaves ample opportunity for discovery of novel approaches and improvements to crop disease management by investigating specific crop diseases within the context of microbial biofilms [18]. 

**Figure 1:**
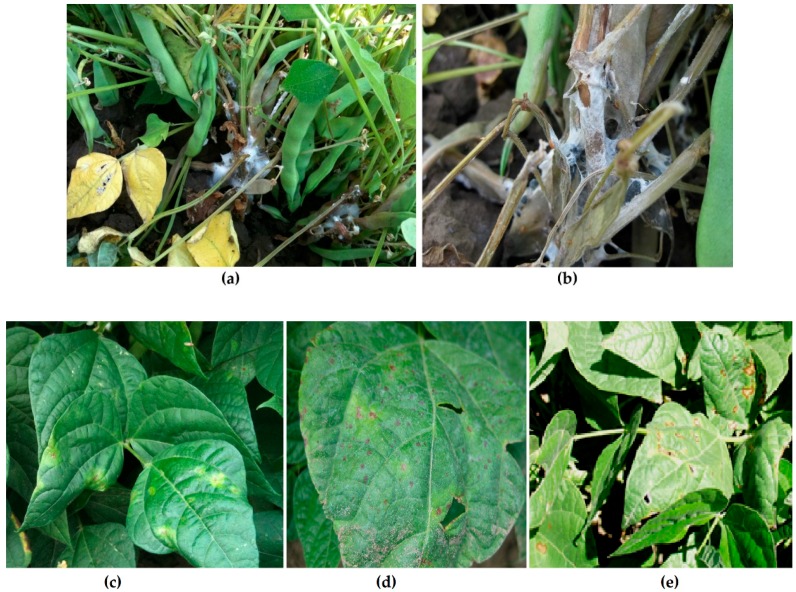
Disease symptoms of white mold (upper panels) and bacterial blights (lower panels). (**a**) Cottony white mycelium of *S. sclerotiorum* at the base of a maturing bean (*Phaseolus vulgaris* L.) plant. (**b**) Networks of cottony, white mycelium, and small black sclerotia can be seen on the dead/dying bean stems and leaves. (**c**) Symptoms of halo blight caused by *Pseudomonas syringae* pv. *phaseolicola.* (**d**) Symptoms of brown spot caused by *Pseudomonas syringae* pv. *syringae*. (**e**) Symptoms of common blight caused by *Xanthomonas axonopodis* pv. *phaseoli*.

*Sclerotinia sclerotiorum* (Lib.) de Bary is a biofilm-forming fungus that causes above ground disease symptoms on many field and horticultural crops. This pathogen is responsible for millions of dollars in crop losses annually [19]. *Sclerotinia sclerotiorum* causes the disease ‘white mold’ (WM) on bean (*Phaseolus vulgaris* L.), and symptoms are shown in Figure 1. The disease is highly destructive and difficult to manage [20]. Another example of a disease on dry bean is the bacterial blight (BB) disease complex caused by *Pseudomonas syringae* pv. *phaseolicola, Pseudomonas syringae* pv. *syringae, Xanthomonas axonopodis* pv. *phaseoli,* and *Curtobacterium flaccumfaciens* pv. *flaccumfaciens*. All these bacteria form biofilms during one or more phases of their disease cycles. BB leads to above ground symptoms of chlorotic and/or necrotic lesions as shown in Figure 1, or wilt symptoms in the case of *Curtobacterium*. While these bacterial diseases are not as devastating as WM, they are still difficult to manage and can cause significant losses [21]. A lack of resistant germplasm to both WM and BB necessitates the use of fungicides and bactericides for management, however adequate disease control may not always be provided by pesticide applications, even when applied as directed by the manufacturer. For example, fungicide applications as recommended during the flowering stage for control of WM can provide significant protection in some years, but not in others. This variability in efficacy is often ascribed to the highly responsive nature of WM severity to weather-related environmental effects [22]. Similarly, some bactericidal seed treatment options have inconsistent results, especially in Canada where streptomycin is no longer approved for use. BBs are notoriously seed-borne, so clean seed programs and seed treatments are foundational tools used to control them. 

The study of biofilms in diseases of humans and animals has revealed another reason for the failure of chemical interventions in clinical and field settings. Failed interventions can frequently be attributed in large part to the fact that biofilms are often more tolerant to chemical treatments than free-floating planktonic cultures typically tested in the laboratory [23,24]. Due to the high degree of tolerance or resistance seen in biofilms compared to the relative susceptibility of planktonic cells, treatments that appear efficacious in a laboratory study of planktonic cells will frequently underperform in clinical and field situations due to the presence of biofilms that are much more recalcitrant to eradication or remediation. 

The purpose of this study was to take what was known from medical and environmental biofilm studies and apply it to control of WM and BB on dry bean. First, the effect(s) of combining commercially available fungicides with metallic cations was examined with screening for improved fungicide efficacy versus *S. sclerotiorum* biofilms. Second, oxidized, high-valency silver compounds were evaluated as seed coatings for inhibitory effects on biofilm formation by BB pathogens. The hypothesis was that the addition of metallic ions as fungicide tank mix partners, or as anti-bacterial coatings, would reduce biofilm populations of these fungal and bacterial phytopathogens. In this study a high throughput, static biofilm reactor platform was utilized to grow the fungal and bacterial biofilms in aseptic, pure cultures. For screening fungicides in combination with metallic cations versus *S. sclerotiorum* biofilm, the high throughput capacity of the MBEC Assay^®^ was used, which allowed for the fungicides and metallic cations to be evaluated in combination at each of three concentrations for a total of 324 unique combinations, performed in quintuplicate. For seed coating experiments, the BEST Assay™ was utilized because it accommodated biofilm formation and surface testing on actual bean seeds. The aim of these experiments was to identify combinations of fungicides and metallic cations that showed potential to improve the control of WM, and to evaluate novel high valency silver compounds as seed coatings to prevent biofilm formation of BB pathogens on dry bean seeds.

## 2. Results

### 2.1. Culturing, Treatment, and Quantification of Microbial Biofilms Using the MBEC Assay^®^ and the BEST Assay™

#### 2.1.1. Fungal Biofilms (*Sclerotinia sclerotiorum*)

**Figure 2:**
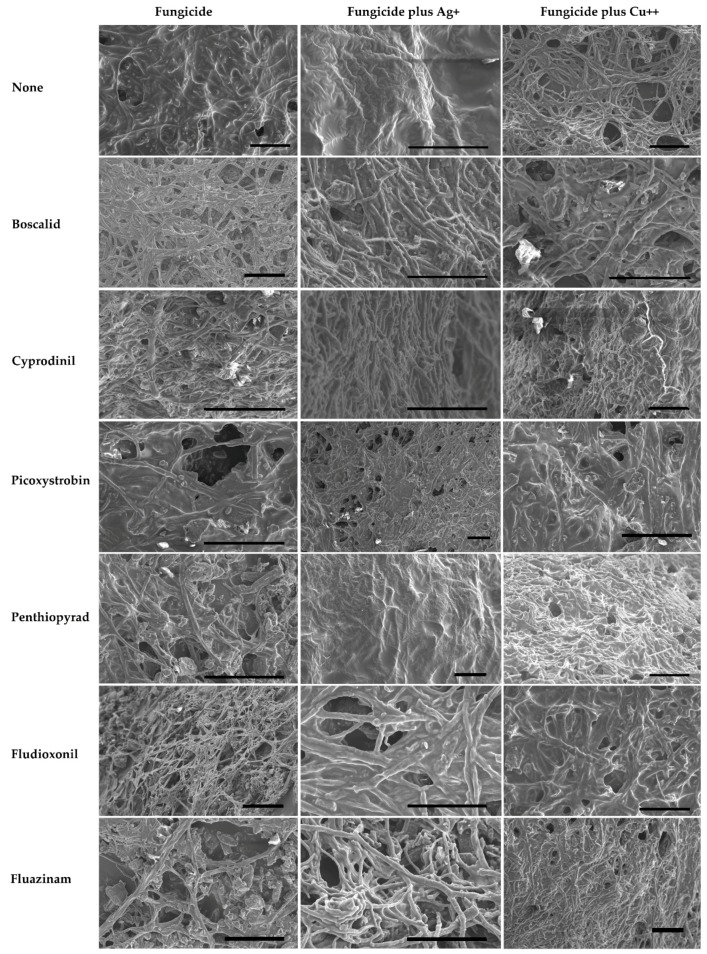
Scanning electron micrographs of *Sclerotinia sclerotiorum* biofilms on cellulose-coated plastic pegs on the MBEC Assay^®^ plate. Extensive colonization of the pegs was seen after 48 h. Hyphae were stacked in multiple layers, were tubular in appearance and were often seen encased in the remaining artifacts of what appeared to be self-produced polymeric matrix (EPS). After exposure to fungicide and fungicide plus metallic cations Ag^+^ or Cu^2+^,some hyphae appeared shrunken or lysed, had a rough external morphology with pitting and sometimes collapsed. Scale bars = 100 µm.

*Sclerotinia sclerotiorum* biofilms covered most of the MBEC Assay^®^ pegs and hyphae were stacked in multiple layers, with evidence of EPS encasing the biofilm (Figure 2, upper left panel). The appearance and arrangement of hyphae, and the morphology of the biofilm, were consistent with those of other filamentous fungal biofilms formed by phytopathogenic fungi on plant and/or biofilm reactor surfaces [12,13]. 

#### 2.1.2. Bacterial Biofilms

**Figure 3:**
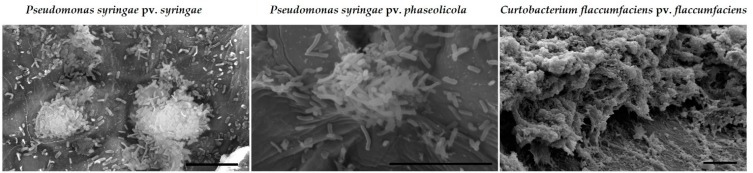
Scanning electron micrographs of biofilms or aggregates formed by three bacterial blight pathogens on the surfaces of dry bean tissues. The biofilms contain layers of bacterial growth and artifacts of the EPS that remains after fixation and dehydration are visible. Scale bars = 10 µm.

Bacterial biofilms have been observed on the surfaces of dry bean seeds formed by the phytopathogenic bacteria *Pseudomonas syringae* pv. *phaseolicola* and *Curtobacterium flaccumfaciens* pv. *flaccumfaciens*. Examples of a biofilms of these bacteria on the surfaces of dry bean are shown in Figure 3. Coatings with silver compounds were evaluated to see if they could prevent the formation of biofilms like those observed in Figure 3.

### 2.2. Fungicide and Metallic Cation Effects on Biofilms Formed by Bacterial Blight and White Mold Pathogens

#### 2.2.1. Fungicides Tank Mixed with Metallic Cations versus *S. sclerotiorum* Biofilms.

Morphological changes in the biofilm were noted after treatments with metals and fungicides. For example, hyphae often appeared shrunken, lysed, or shriveled after treatment with metal ions and/or fungicides. Exposure to fungicides and metals also appeared to cause pitting or swelling of hyphal cell walls (Figure 2). 

**Figure 4:**
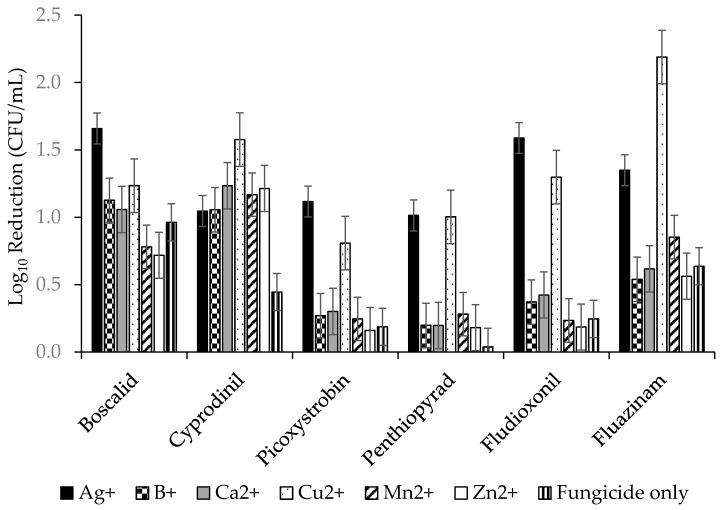
Log_10_ (Lg) reductions in CFU/mL for six fungicides in combination with six metals. Error bars represent the standard error of the mean.

To determine which individual combinations were best at improving efficacy, all fungicide x metal combinations were compared. The ANOVA gave an R^2^ = 65.29% with *p* ≤ 0.00 for variance between fungicides, metals and interactions of fungicides and metals. The results are shown graphically in Figure 4 and the mean separations for all are shown in supplementary Table S1. Ag^+^ was best with boscalid, picoxystrobin, and fludioxonil, and Ag^+^ and Cu^2+^ were equally good at improving the efficacy of penthiopyrad, while cyprodinil and fluazinam had the greatest improvement with Cu^2+^. Cyprodinil was the only fungicide that saw a significant improvement with the addition of Zn^2+^. 

A second ANOVA, using only the top four fungicides (boscalid, cyprodinil, fludioxonil, and fluazinam), was performed to reduce the size of the table. The ANOVA gave an R^2^ = 60.71% with *p* ≤ 0.003 for variance between fungicides, metals and fungicide/metal interactions. Mean separations using Tukey’s pairwise comparisons for these 24 combinations are shown in Table 1.

#### 2.2.2. Abilities of Silver-Based Coatings to Reduce Adherence of Bacterial Blight Pathogens

**Figure 5:**
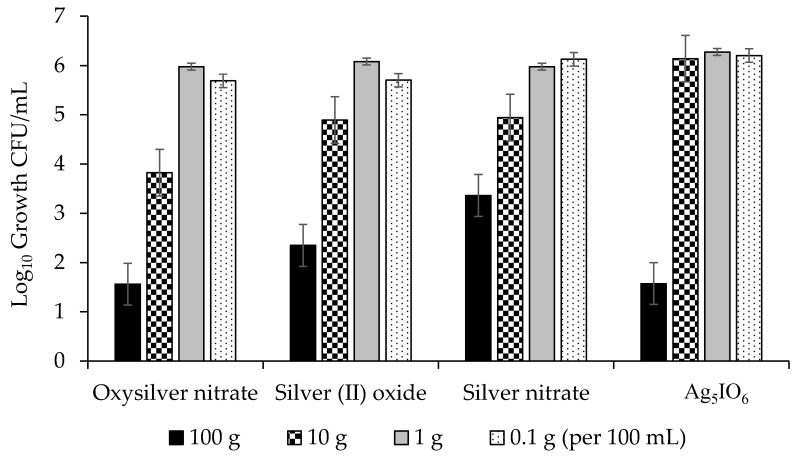
Effects of four silver-based seed coatings on the lg growth (CFU/mL) of *Pseudomonas syringae* pv. *syringae*.

**Figure 6:**
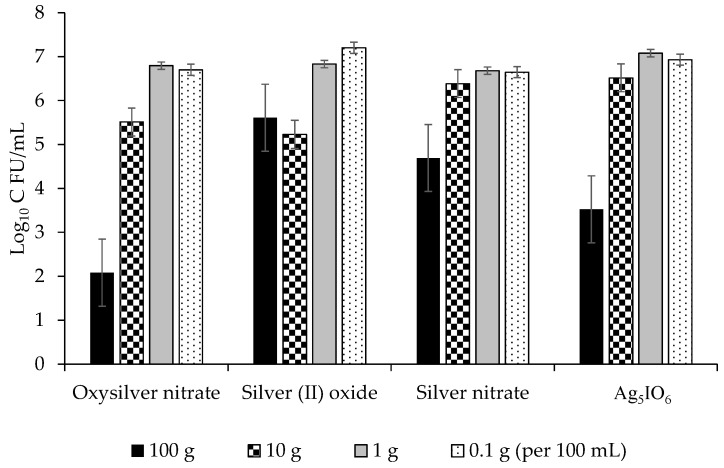
Effects of four silver-based seed coatings on the lg growth (CFU/mL) of *Pseudomonas syringae* pv. *phaseolicola*.

**Figure 7:**
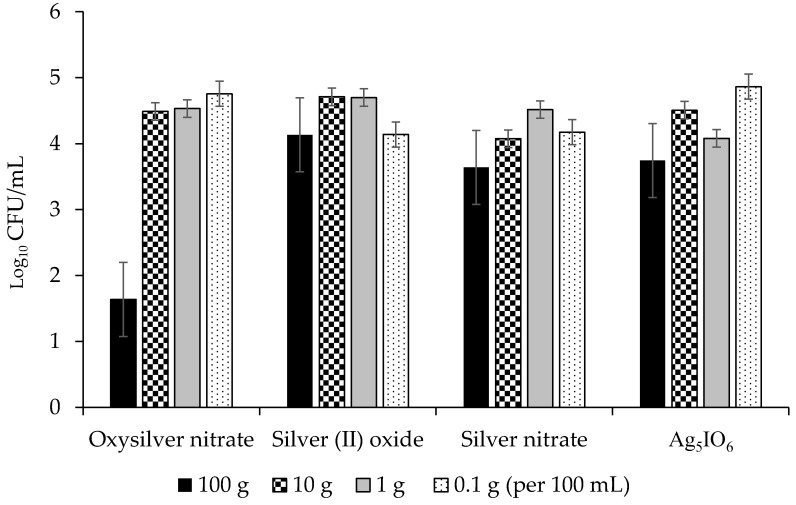
Effects of four silver-based seed coatings on the lg growth (CFU/mL) of *Curtobacterium flaccumfaciens* pv. *flaccumfaciens*.

The highest concentrations were generally the most effective for all four silver coatings and were able to reduce growth of *Pseudomonas syringae* pv. *syringae* by up to 5 lg, or 99.999% (Figure 5). Of the three bacteria evaluated, *P. syringae* pv. *syringae* was most sensitive to the silver coatings followed by *P. syringae* pv. *phaseolicola*, and *C. flaccumfaciens* pv. *flaccumfaciens* was the most tolerant (Figure 5, Figure 6 and Figure 7). All data points for a given coating were pooled and ANOVA performed. The R^2^ was 76.93% and significant differences were observed between concentrations and coatings at *p* ≤ 0.031. Oxysilver nitrate was the most effective treatment and was significantly better than the other silver coatings except versus *P. syringae* pv. *syringae* where it was equal to silver (II) oxide and Ag_5_IO_6_, but better than silver nitrate (Table 2). 

## 3. Discussion

It is now well-known that microorganisms grow in natural settings as biofilms. It has also been known for many years that copper and silver ions have antimicrobial properties effective against microbial biofilms. Despite this readily available knowledge, a biofilm approach to crop disease management has only recently been adopted [18], and tank mixing fungicides with metallic ions is still relatively novel [25]. Furthermore, there is still much to learn regarding how metallic ion coatings may aid in preventing or remediating plant diseases. This study hypothesized that a biofilm approach to fungal and bacterial diseases on dry beans could lead to novel solutions, and that metallic cations may improve fungicide efficacies and provide effective coatings that prevent or reduce bacterial biofilm formation on seed. 

### 3.1. Fungicide Efficacies for Management of Sclerotinia sclerotiorum (White Mould) Biofilms

Sclerotinia stem rot and white mold are very economically important diseases in production of crops such as dry beans and canola [20,26]. Fungicides are important management tools because of the lack of genetic resistance, and inability of cultural practices to manage the diseases. Therefore, understanding the efficacies of fungicides and working toward incremental improvements in their performance are important goals. Fungicide efficacy is most commonly measured using amended-agar assays to determine EC_50_. For example, a number of studies have reported the EC_50_’s for fungicides used to manage *S. sclerotiorum* [27,28,29]. While this information is useful and important, it may overestimate the efficacies of fungicides because they were tested against cultures growing on a nutrient rich agar gel, and not as a biofilm. Testing fungicide efficacy versus biofilms frequently reveals that biofilms are much more tolerant of chemical treatments [18,30]. The results of this study confirmed that biofilms were more difficult to treat with fungicides as concentrations between 0.67 and 4.9 µg/mL did not provide significant control and were all unable to achieve 1 lg reductions (90%) in fungal populations, whereas reports using the amended-agar approach showed 70% to 98% control concentrations of 0.1 µg/mL [29] and EC_50_ values between 0.059 and 0.085 µl/mL.

### 3.2. Improvement in Fungicide Efficacy via Tank Mixing Metallic Cations

Both positive and negative effects of metallic cations on biofilms have been reported [31,32,33,34,35]. Only recently have some authors demonstrated additive and/or synergistic interactions between copper or silver ions when combined with fungicides [25,36,37]. This study provides the first evidence of metallic ions improving efficacies of fungicides versus *S. sclerotiorum* biofilms. All six fungicides were improved by one or more of the metallic cations. All improvements were statistically significant, except for boscalid which was the most effective fungicide on its own (Figure 4; Table 1). Cu^2+^ and Ag^+^ were the most effective tank mix partners and had a dramatic effect on efficacy (Figure 4). It is acknowledged that much of the improvement in efficacy is due to the direct fungicidal effects of Cu^2+^ and Ag^+^, and some of the morphological changes seen in the *S. sclerotiorum* biofilms, such as pitting in cell walls, were similar to those described for silver nanoparticles [34]. However, some of the improvements in efficacy are greater than the sum of the two actives. For example, fluazinam was able to achieve a 0.64 lg reduction in CFU/mL, but with Cu^2+^ added, it gave a 2.18 lg reduction. Going from approximately half a lg reduction to greater than 2 lg reduction is more than just the sum of the activities of the two compounds and shows a highly synergistic interaction. Additive and synergistic improvements between metallic cations and antibiotics or fungicides have been reported [38,39,40,41]. In some cases, the improvements are simply the additive sum of direct actions of both partners, but others are greater than the sum of their parts. These synergistic interactions have been hypothesized to be due to the stabilization of the antibiotic or fungicide as it forms a complex with the metal, or the permeabilization of the microbial cells by the metal ions that potentiate the fungicide’s activity. 

This study provided the first evidence that *S. sclerotiorum* biofilms are more difficult to eradicate than planktonic cultures in broths or semi-solid agar gels. It also showed for the first time the potential role of metallic cations for improvement of fungicide efficacy at lower doses. This is especially important for *S. sclerotiorum,* because this fungus is known to generate new genetic variation via increases in mutation within clonal populations as a result of exposures to sub-lethal doses of fungicides [42]. Fungicide insensitivity is a major concern for plant pathosystems, like white mold/dry bean and stem rot/canola, where current practice relies heavily on fungicides to prevent yield and quality losses. Improvements in fungicide efficacy while maintaining lower doses is more environmentally responsible and allows judicious fungicide use programs that can avoid unnecessary development fungicide insensitivity.

### 3.3. Silver Coatings for Prevention of Bacterial Blight on Dry Bean Seed

Bacterial blights on dry bean are important disease issues for bean producers. Clean seed programs and seed treatments are critical management techniques to prevent bacterial blight epidemics but are not always effective due to asymptomatic seed infections and the lack of bactericidal seed treatment options in some jurisdictions [43]. Furthermore, genetic resistance is not available in early-maturing, high-yielding varieties. Novel approaches to bacterial blight management are needed to deal with these diseases.

The use of metallic ions as bactericides is not new, but metallic ion seed coatings for prevention of disease is not common. Copper-based seed treatments have been used for this purpose, but in some cases the rate needed for efficacy was near the threshold of crop injury, and those treatments that do not cause crop injury are inconsistent in their effectiveness (M. Harding, unpublished). This study demonstrated that silver coatings can reduce adherence of bacterial blight pathogens on dry bean seed surfaces. Oxysilver nitrate was the best silver-based coating for reducing populations on dry bean seed, and it reduced populations by over 5 lg (99.999%) at the highest concentration used. It was effective against all three bacterial blight pathogens evaluated (Figure 5, Figure 6 and Figure 7). Ag_5_IO_6_ was the second most effective silver-based coating. This activity was observed after 24 h exposure to soil, indicating that the treatments could have continued efficacy post-planting. These compounds are easy to manufacture and may be candidate tools for reducing seed-transmitted and seed-borne bacterial blights in dry edible bean.

### 3.4. General Conclusions

This study has taken a biofilm approach to screen for improved control of fungal and bacterial diseases on dry bean. For the fungal disease white mold, novel combinations of fungicides and metallic cations were evaluated using the MBEC Assay^®^. Ag^+^ and Cu^2+^ were able to improve fungicide efficacies significantly, and some evidence of synergy was observed for fluazinam plus Cu^2+^. The authors wish to caution readers on the potential dangers of combining fungicides with metallic cations in non-approved, unregistered or off-label combinations. The results presented in this study should not be considered safe or appropriate for commercial use. Always follow pesticide label and SDS recommendations for use. 

For bacterial diseases, silver-based seed coatings were evaluated using a modified BEST Assay^®^ to discover those that could prevent or reduce colonization of dry bean seeds by three phytopathogenic bacteria. Oxysilver nitrate was consistently best at reducing bacterial biofilm formation. The potent anti-biofilm potential of this compound makes it an ideal candidate for use as an antibacterial seed treatment for control of seed-borne or seed-transmitted bacterial diseases. Additionally, the potential of oxysilver nitrate may extend beyond the three bean blight diseases tested, and may offer solutions to seed-borne bacterial diseases plaguing crops other than dry bean.

The experimental models employed in this study successfully identified a number of possible solutions to the microbial disease issues faced by dry bean producers. However, it is important to note that every model and method has limitations [44]. For example, the MBEC Assay^®^ and BEST Assay^™^ are static reactors rather than continuous flow, and as a result nutrient limitation becomes an increasing issue with time. Additionally, the size of each experimental unit is limited to the size of a microtiter plate well. Therefore, while this model may approximate disease management of biofilms at a commercial scale, the results may be specific to the model, and may not translate well to the commercial scale. Further testing will be required to validate these results in the field. Despite these limitations, the model and methodology can be very useful when experiments have many treatments or require a high throughput approach. Another benefit is that this approach is not limited to phytopathology, but can be applied to human and animal pathosystems involving bacterial or fungal biofilms. Additionally, it could be used to investigate and solve issues of fungicide insensitivity in human, animal, and plant pathosystems.

## 4. Materials and Methods

### 4.1. Culturing, Challenge, and Recovery of Fungal Biofilms

*Sclerotinia sclerotiorum* biofilms were grown in Yeast Mannitol Broth (YMB) pH = 7.4 using the MBEC Assay^®^ as described by Harding et al. [44]. Six fungicides were evaluated; boscalid, cyprodinil, fludioxonil, fluazinam, penthiopyrad, picoxystrobin and six metallic cations were evaluated; Ag^+^, B^+^, Ca^2+^, Cu^2+^, Mn^2+^, and Zn^2+^. The fungicides and metallic cations were prepared at three concentrations (Table 3). Each of the fungicide concentrations were mixed separately with each of the metal cation concentrations for a total of 324 treatment combinations. 

Biofilm survival was quantified by detection of live cells using a Resazurin cell viability assay (Sigma-Aldrich, Oakville, ON, Canada) according to manufacturer’s protocol. Briefly, a standard curve was created using a serial dilution of a concentrated culture that had been quantified using a hemocytometer. To each dilution, 100 µL of resazurin solution was added and read at A_595_ (λ_max_ for Resazurin). From this, the linear equation of absorbance vs. CFU was calculated. The extinction for resazurin coefficient was calculated from the data in the linear region of the graph. Biofilms were exposed to the fungicide for 24 h in 300 µM resazurin. After the fungicide treatment, quantification was done using a microplate reader at 595 nm. The lg recovery was calculated as:

$$Log\;Recovery=\ln\left(\frac{A595}{0.0018}\right)/1.190$$

The lg reduction of cells within the biofilm was calculated as:

Lg recovery (growth control) − lg recovery (test).

The samples were fixed using 5% glutaraldehyde in 0.1 M sodium cacodylate buffer, pH 7.5 for 24 h, then let air dried for 72–96 h. The samples were carefully affixed to the appropriately labelled aluminum stubs using double-sided disc of carbon tape, and gently pressed to fix specimens securely. The non-conducting samples were sputter coated with a conductive layer (Platinum-Gold) to improve imaging before capturing the SEM images using Hitachi S3700N Scanning Electron Microscope (Hitachi High Technologies, Rexdale, ON, Canada)).

### 4.2. Testing Silver Coatings for Prevention of Bacterial Blight on Dry Bean Seed

Dry bean seeds were rinsed briefly with sterile water and air-dried in sterile Petri dishes with the lids ajar in a sterile environment (biosafety cabinet). The seeds were then sterilized by UV irradiation by continuous exposure to UV light for 36 h. 

Silver solutions were prepared according to Table 4. The most concentrated solutions, suspensions and slurries were prepared in sterile distilled water by stirring, vortexing, or shaking for 15 min (maximum), protected from light. Dilutions were performed to create the lower concentrations while mixing thoroughly to ensure a consistent slurry/suspension/solution, and were also protected from light. Silver nitrate formed a solution, but oxysilver nitrate, silver (II) oxide and Ag_5_IO_6_ did not fully dissolve. Coatings were applied by placing 15 g of seed (~35 beans) in two 50 mL Falcon tubes, adding 75 µL of solution/slurry to the seeds per tube and then rotating tubes for 5 min. The seeds were allowed to air dry with minimal handling in the dark in a laminar flow hood, and then were stored with desiccant at 4 °C in the dark until tested. 

Coated seeds were aseptically secured with sterile forceps onto lids of appropriately shaped BEST Assay^TM^ 12 well plates using a thermoplastic adhesive. All seeds extended approximately the same distance from the lid and the seeds extended sufficiently far from the lid that most of the seed was in contact with the solution in the well beneath, but the adhesive was not. Each well (except sterility controls) was filled with 400 ± 50 mg of soil (a greenhouse professional growing mix) and 3 mL sterile water, with stirring. The lids with attached seeds were placed into the soil-containing wells for 24 h, then rinsed in wells containing 3.5 mL sterile water prior to inoculation, to mimic the effect that soil contact would have on the treatments. Microorganisms used: *Pseudomonas syringae* pv. *syringae* (8728a), *Pseudomonas syringae* pv. *phaseolicola* (HB9), and *Curtobacterium flaccumfaciens* pv. *flaccumfaciens* (wild type). All three microorganisms were grown at 25 ± 2 °C on Brain Heart Infusion (BHI) agar. The *Curtobacterium* was grown in BHI broth, while the *Pseudomonads* were grown in Potato Dextrose Broth (PDB) + 1% special peptone. The BEST Assay™ lids with the attached seeds were placed in wells containing 3.5 mL of full-strength inoculum (grown for 24 h at 150 rpm and vortexed, ~10^9^ CFU/mL) for 10 min, then placed over empty plates and incubated for 1 h at room temperature in a humid environment to allow the microbes to attach to the seeds. The lids with attached seeds were then placed in wells containing 3.5 mL of sterile water for 30 min to allow the treatments time to act on the adhered microbes. Recovery and calculations were performed as described in Harding et al. [45] using DE neutralizer as the recovery solution.

## 5. Patents

Olson ME, Nadworny PL, Omar AM, Cabrera YE. Family of silver(I) periodate compounds having broad microbial properties. Issued 08/08/2017. US Patent No. 9,723,843.

## Figures and Tables

**Table 1:** Lg reduction in colony forming units (CFU)/mL in *Sclerotinia sclerotiorum* biofilms for combinations of four fungicides with six metallic cations. Means that do not share a letter in common are significantly different at *p* ≤ 0.003.

Fungicide	Metal	Lg Reduction (CFU/mL)
Fluazinam	Cu^2+^	2.18 ^a^
Boscalid	Ag^+^	1.66 ^ab^
Fludioxonil	Ag^+^	1.59 ^ab^
Cyprodinil	Cu^2+^	1.58 ^abc^
Fluazinam	Ag^+^	1.35 ^abcd^
Fludioxonil	Cu^2+^	1.30 ^abcde^
Boscalid	Cu^2+^	1.23 ^bcde^
Cyprodinil	Ca^2+^	1.23 ^bcde^
Cyprodinil	Zn^2+^	1.21 ^bcde^
Cyprodinil	Mn^2+^	1.17 ^bcdef^
Boscalid	B^+^	1.13 ^bcdefg^
Boscalid	Ca^2+^	1.06 ^bcdefg^
Cyprodinil	B^+^	1.05 ^bcdefg^
Cyprodinil	Ag^+^	1.05 ^bcdefg^
Boscalid	None	0.96 ^bcdefg^
Fluazinam	Zn^2+^	0.85 ^bcdefg^
Boscalid	Mn^2+^	0.78 ^bcdefg^
Boscalid	Zn^2+^	0.72 ^bcdefg^
Fluazinam	None	0.64 ^cdefg^
Fluazinam	Ca^2+^	0.62 ^defg^
Fluazinam	Zn^2+^	0.56 ^defg^
Fluazinam	B^+^	0.54 ^defg^
Cyprodinil	None	0.44 ^defg^
Fludioxonil	Mn^2+^	0.43 ^defg^
Fludioxonil	Ca^2+^	0.42 ^defg^
Fludioxonil	B^+^	0.37 ^efg^
Fludioxonil	None	0.25 ^fg^
Fludioxonil	Zn^2+^	0.19 ^g^

**Table 2:** Mean lg CFU/mL values for three bacterial blight pathogens on seeds with one of each of four silver coatings. Means that do not share a letter in common are statistically significantly different according to Tukey’s pairwise comparison at *p* ≤ 0.031.

Silver Coating	PSS ^1^ Growth (lg CFU/mL)	PSP ^2^ Growth (lg CFU/mL)	CFF ^3^ Growth (lg CFU/mL)
Oxysilver nitrate	4.26 ^a^	5.27 ^a^	3.85 ^a^
Silver (II) oxide	4.75 ^ab^	6.22 ^b^	4.42 ^b^
Ag_5_IO_6_	5.05 ^ab^	6.10 ^b^	4.10 ^b^
Silver nitrate	5.1 ^b^	6.01 ^b^	4.30 ^b^

^1^ PSS = *Pseudomonas syringae* pv. *syringae*. ^2^ PSP = *Pseudomonas syringae* pv. *phaseolicola*. ^3^ CFF = *Curtobacterium flaccumfaciens* pv. *flaccumfaciens*.

**Table 3:** Fungicides and metallic cations used in this study.

Compound	Source	Concentrations (g/L)
Boscalid	2-chloro-*N*-[2-(4-chlorophenyl)phenyl]pyridine-3-carboxamide	4.9, 2.7, 1.79
Cyprodinil	4-cyclopropyl-6-methyl-*N*-phenylpyrimidin-2-amine	2.74, 1.83, 1.0
Fluazinam	3-chloro-*N*-[3-chloro-2,6-dinitro-4-(trifluoromethyl)phenyl]-5-(trifluoromethyl)pyridin-2-amine	4.55, 3.03, 1.67
Fludioxonil	4-(2,2-difluoro-1,3-benzodioxol-4-yl)-1*H*-pyrrole-3-carbonitrile	1.83, 1.22, 0.67
Penthiopyrad	1-methyl-*N*-[2-(4-methylpentan-2-yl)thiophen-3-yl]-3-(trifluoromethyl)pyrazole-4-carboxamide	2.72, 1.49, 0.99
Picoxystrobin	methyl (*E*)-3-methoxy-2-[2-[[6-(trifluoromethyl)pyridin-2-yl]oxymethyl]phenyl]prop-2-enoate	2.0, 1.1, 0.73
Ca^2+^	Ca(NO_3_)_2_	0.029, 0.015, 0.0096
B^+^	Na_2_[B_4_O_5_(OH)_4_]	3.05,1.68, 1.12
Cu^2+^	CuSO_4_,	3.05, 1.68, 1.12
Mn^2+^	MnSO_4_	3.05, 1.68, 1.12
Ag^+^	AgNO_3_	4.55, 2.5, 1.67
Zn^2+^	ZnSO_4_	5.09, 2.8, 1.86

**Table 4:** Silver solutions used in this study.

Compound	Formula	Concentrations * (mg/1.5 mL)
Oxysilver nitrate	Ag(Ag_2_O_4_)_2_NO_3_	150, 15, 1.5, 0.15
Silver (II) oxide	AgO	138, 13.8, 1.38, 0.138
Silver nitrate	AgNO_3_	189, 18.9, 1.89, 0.189
Pentasilver hexaoxoiodate	Ag_5_IO_6_	169, 16.9, 1.69, 0.169

* These concentrations result in equivalent total silver contents per treatment.

## Supplementary Materials

The following are available online; Table S1: Lg reductions in CFU/mL for each fungicide and for interactions between fungicide/metallic cation combinations.
