# Supplementary material for: Improved Methods for Treatment of Phytopathogenic Biofilms: Metallic Compounds as Anti-Bacterial Coatings and Fungicide Tank-Mix Partners
Source: Molecules. 2019 Jun 22;24(12):2312. doi: 10.3390/molecules24122312 (PMC6630349; doi:10.3390/molecules24122312)
Supplement: Supplementary file 1 [file molecules-24-02312-s001.pdf]

molecules

MDPI

Article

Improved methods for treatment of phytopathogenic biofilms: metallic compounds as anti-bacterial coatings and fungicide tank-mix partners.

Michael W. Harding [1,*], Patricia Nadworny [2,*], Brenton Buziak [2], Amin Omar [2], Greg C. Daniels[1], and Jie Feng [3]

1 Alberta Agriculture and Forestry, Crop Diversification Centre South, 301 Horticulture Station Road East, Brooks, Alberta, T1R 1E6, Canada; michael.harding@gov.ab.ca
2 Innovotech, Inc.; Suite 101, 2011-94 Street, Edmonton, Alberta, T6N 1H1, Canada; info@innovotech.ca
3 Alberta Agriculture and Forestry, Alberta Plant Health Lab, 17507 Fort Road NW, Edmonton, Alberta, T5Y 6H3, Canada; planthealthlab@gov.ab.ca
* Correspondence: michael.harding@gov.ab.ca (M.W.H.); patricia.nadworny@innovotech.ca (P.N.); Tel.: +1-403-362-1338 (M.W.H.)

Supplementary data

Table S1. Log_{10} reductions in CFU/mL for each fungicide and fungicide x metallic cation combination. Means that do not share a letter in common are statistically significantly different according to Tukey's pairwise comparison (p=0.000).

Fungicide	Metal	Log_{10} Reduction (CFU/ml)
Fluazinam	Cu^{2+}	2.19 a
Boscalid	Ag^{+}	1.65 ab
Fludioxonil	Ag^{+}	1.59 abc
Cyprodinil	Cu^{2+}	1.58 abc
Fluazinam	Ag^{+}	1.35 abcd
Fludioxonil	Cu^{2+}	1.30 abcde
Boscalid	Cu^{2+}	1.23 bcdef
Cyprodinil	Ca^{2+}	1.23 bcdef
Cyprodinil	Zn^{2+}	1.21 bcdef
Cyprodinil	$Mn2^{+}$	1.17 bcdefg
Boscalid	B^{+}	1.13 bcdefgh
Picoxystrobin	Ag^{+}	1.12 bcdefgh
Boscalid	Ca^{2+}	1.06 bcdefghi
Cyprodinil	B^{+}	1.05 bcdefghi
Cyprodinil	Ag^{+}	1.05 bcdefghi
Penthiopyrad	Ag^{+}	1.01 bcdefghi
Penthiopyrad	Cu^{2+}	1.00 bcdefghi
Boscalid	None	0.96 bcdefghi
Fluazinam	Mn^{2+}	0.85 bcdefghij
Picoxystrobin	Cu^{2+}	0.81 bcdefghij
Boscalid	Mn^{2+}	0.78 bcdefghij
Boscalid	Zn^{2+}	0.72 cdefghij
Fluazinam	None	0.64 defghij
Fluazinam	Ca^{2+}	0.62 defghij
Fluazinam	Zn^{2+}	0.56 defghij
Fluazinam	B^{+}	0.54 defghi
Cyproinil	None	0.44 defghij
Fludioxonil	Mn^{2+}	0.43 efghij

Fludioxonil	Ca^{2+}	0.42 efghij
Fludioxonil	B^{+}	0.37 fghij
Picoxystrobin	Ca^{2+}	0.30 ghij
Penthiopyrad	Mn^{2+}	0.28 ghij
Picoxystrobin	B^{+}	0.27 ghij
Picoxystrobin	Mn^{2+}	0.25 hij
Fludioxonil	None	0.25 hij
Penthiopyrad	B^{+}	0.20 ij
Penthiopyrad	Ca^{2+}	0.20 ij
Picoxystrobin	None	0.19 ij
Fludioxonil	Zn^{2+}	0.19 ij
Penthiopyrad	Zn^{2+}	0.18 ij
Picoxystrobin	Zn^{2+}	0.16 ij
Penthiopyrad	None	0.04 j
